# A Realist Synthesis of Interprofessional Collaboration in the Early Years; Becoming Familiar with Other Professionals

**DOI:** 10.5334/ijic.5482

**Published:** 2020-09-28

**Authors:** Ruben Fukkink, Esmée Soraya Lalihatu

**Affiliations:** 1 Centre for Applied Research in Education (CARE), Amsterdam University of Applied Sciences, Amsterdam, NL; 2 Research Institute of Child Development and Education, University of Amsterdam, Amsterdam, NL

**Keywords:** interprofessional collaboration, realist review, early childhood education and care (ECEC), community setting

## Abstract

Research into interprofessional collaboration (IPC) has predominantly focused on health care and specialized care settings, but there is an increasing interest in interprofesssional ‘teams around the child’ in community-based settings. We conducted a realist synthesis of empirical studies into IPC between youth professionals, often in regular community settings, to explore barriers and facilitators of IPC. Included studies were coded with an elaborated scheme to chart the focus of studies and to identify moderators and context-mechanism-outcome configurations of IPC. Professional and normative integration was the main focus of the included studies. Most studies emphasized the challenges of IPC in practice, like unclear roles of self and others, lack of trust and inadequate communication. Other perceived barriers are excluding others in the planning of interventions, taking ownership of plans (vs. sharing) and different modes of communication. Interprofessional education, co-location of staff, acting as a mediator in the team, organising formal and informal meetings, conflict resolutions, self-sacrifice, and conceptualizing practice were perceived as facilitators of IPC. Future IPC research into community-based settings should include all professional stakeholders and the children and their families to evaluate outcomes at both interprofessional and clinical level.

## Introduction

The transformation of autonomous and often fragmented children’s services into an integrated service is a topical issue in several countries [1,2,3]. The trend to offer more comprehensive youth services is stimulated by inclusive education policies, which aim to integrate children with special needs in mainstream childcare and school settings with the help of special staff. An integrated service with professionals from various disciplines who collaborate in the assessment and monitoring, providing support, health education, or referral [4] may be helpful in the early identification and treatment of children’s behavioural problems and developmental delays. In some countries, multi-professional teams already operate in community-based settings, like childcare and schools, but integrated community services for children and their families are certainly not self-evident [5,6].

Transforming the workforce in regular community-based care brings together different professionals (e.g., caregivers, teachers, social workers, nurses and youth care specialists) from various sectors, including early childhood education and care, primary schools, social work and youth care. This interprofessional collaboration in a ‘team around the child’ has only a relatively brief history, acknowledging differences between countries. IPC is certainly not self-evident for many individual practitioners, the interprofessional teams and their organisations which aim ‘to meet special needs in ordinary schools’, as Hanko (1986) [7] put it concisely. However, there is a growing interest in interprofessional teams in community-based services for youth [8,9].

Reviews of IPC, which predominantly relate to health care, have revealed a number of important barriers and facilitators of IPC [10,11,12,13,14,15,16]. Some studies that explored the relatively new territory of children’s services, have also suggested factors that foster or hinder effective IPC. At individual level, working experience [10] and interprofessional education with cross-service training may contribute to fruitful collaboration [17,18]. At team level, regular meetings, mutual trust, clarity about one’s own and others’ professional roles [7,19] and shared team goals [20] contribute to open communication and shared decision making. Conversely, power imbalances may be barriers for interprofessional communication and professional relationships [21,22]. At organisational level, IPC is facilitated by the support of the management [20]. It should be noted, however, that the setting of regular child services is different from health care services related to IPC. IPC does not have a long tradition in regular child services, neither in practice nor in research [3,6]. Depending on the national or regional context, childcare, primary school, youth care, social work and health care may have relatively autonomous positions and cooperation is usually carried out on an ad hoc basis. Relatedly, specialized knowledge of infant mental or physical health, the early diagnosis and treatment of developmental disorders, and interdisciplinary collaboration (e.g., social work, healthcare) are not standard components of the curriculum for staff in early childhood education and care and primary school. Pre-service training for these professions mainly focuses on stimulating children’s development in a broad spectrum with an emphasis on the normal growth and development of young children. The generalization of IPC findings across professional settings is thus not straightforward. In fact, various authors have emphasized that IPC takes shape in an interplay with the professional context, including individual staff, the interprofessional team and the organisational setting. Hence, research should pay close attention to this context to increase our insights into how IPC emerges in different sectors [23,24,25]. Seen from this contextualized perspective, we know yet relatively little about how individual professionals in an interdisciplinary setting collaborate as a team towards an integrated range of children’s services [26,27].

### Present Study

In this study, we focus on the following research question: What are barriers and facilitators of IPC for professionals in community settings for children at individual and team level? We aim, by way of a realist synthesis [28,29,30] to explore which mechanisms may promote or hinder IPC to provide guidance about effective teamwork strategies in community-based children’s services.

## Method

Different traditions of integrating findings from studies exist [23,32]. We chose to integrate findings by way of a realist review, which is a formal qualitative synthesis to evaluate a complex practice like IPC. Realist reviews, which has already been conducted in studies of IPC in health settings for clients with often complex care needs [33], take the contextual dependency explicitly into account and seems also suited in analyzing IPC practice for our domain. A realist review takes into account that specific characteristics of IPC (e.g., co-location of staff) may trigger different responses from professionals (e.g., mutual trust among team members) depending on the specific context (for example, this occurs only if there are regular face-to-face meetings and support from the management). This type of review offers thus a contextualized approach and aims to explore whether a complex practice, like IPC, works in what circumstances. In addition, a realist review allowed us to synthesize the empirical findings from studies with a diversified approach in terms of methodology (i.e., quantitative and qualitative) and theoretical frameworks (i.e., conflict management, role theory).

### Search and Selection of Studies

**Figure 1:**
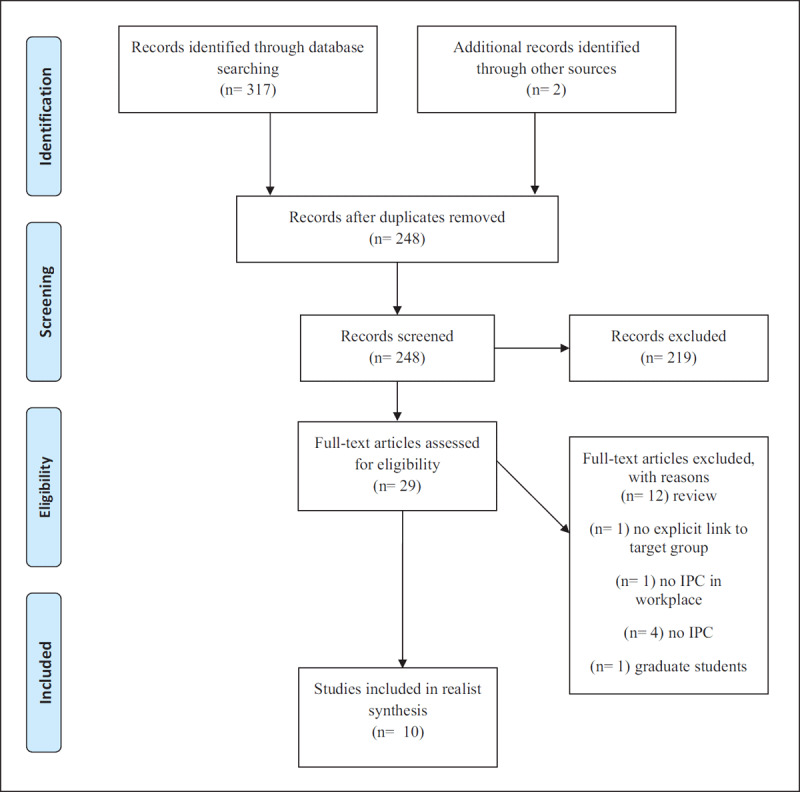
PRISMA Flow Chart of Study Selection.

We searched the databases PsychINFO, Web of Science and Medline (final search at 17^th^ of March, 2019) with an extensive search profile with key words related to interprofessional collaboration (e.g., IPC, multidisciplinary), professionals and their sectors (e.g., child care, youth care) and target population (e.g., toddler, youth) (see Appendix A). Iterative searches were performed in these databases, resulting in 317 references and 248 articles after removal of duplicates (see Figure 1). The second author reviewed the titles and abstracts for relevance based on the inclusion criteria. From the 248 articles, 29 were selected for assessment of the full-text eligibility. We subsequently excluded 19 studies from our analysis that did not report empirical findings but were conceptual or were narrative reviews, leaving 10 primary studies [33,34,35,36,37,38,39,40,41,42]; the results of some studies are integrated in the discussion.

### Coding of Studies

All studies were coded with an extended coding scheme with four layers (see below). We describe our coding below, following the guidelines from Wong and colleagues [43,44].

**General variables: study design and professional background.** We coded a number of methodological and general IPC variables for description of the studies, including the type of design (correlational; cross-sectional; longitudinal; experimental; other); type of study (quantitative, qualitative, combination, or study is explicitly designed as a mixed methods study); sample size; study focuses on child, parent, practitioner and/or management; and the focus on the interaction between these stakeholders (e.g., child- practitioner; parent- practitioner; practitioner-practitioner; management-practitioner); type of measure(s) (interview, survey, focus group, observation, other); study participants: professionals, children and/or parents [45]. Further, we coded the sector (childcare, school, social work, youth care, other) and type of care (primary care in a community service or special care for a specific target group), and the roles of IPC [4,10]: supportive and nurturing; health education and promotion; resources and referral; assessment and monitoring.

**Barriers and facilitators of IPC.** We also coded the studies for a number of key variables that have received meta-analytic support from previous (health) research [10,11,12,13,14,15,16]. We coded the following facilitators: trust/positivity (κ = 1), adequate support (1), leadership (1), high levels of professional development (1), agreement about professional roles (1), clarity about stakeholders (1), clarity about tasks (1), job autonomy (1), adequate job demands (1), no job stress (1), adequate interprofessional education (1) and adequate communication (.62). The corresponding barriers were the same variables ‘reversed’: lack of trust/negativity (κ = 1), lack of support (1), lack of leadership (1), low levels of professional development and IPC skills (1), ambiguity about roles (1), ambiguity about stakeholders (1), ambiguity about tasks (.78), lack of job autonomy (1), high job demands (1), high job stress (1), no or inadequate interprofessional education (1) and lack of communication (1). In Figure 3, facilitators and barriers are indicated with green and red, respectively. After a first pilot and adjustment of the initial scheme, studies were coded independently by the first and second author and inter-coder agreement was determined for each category by using Cohen’s kappa (κ) for nominal variables. All codings, both convergent and divergent ones, were finally discussed between the two coders.

**Figure 2:**
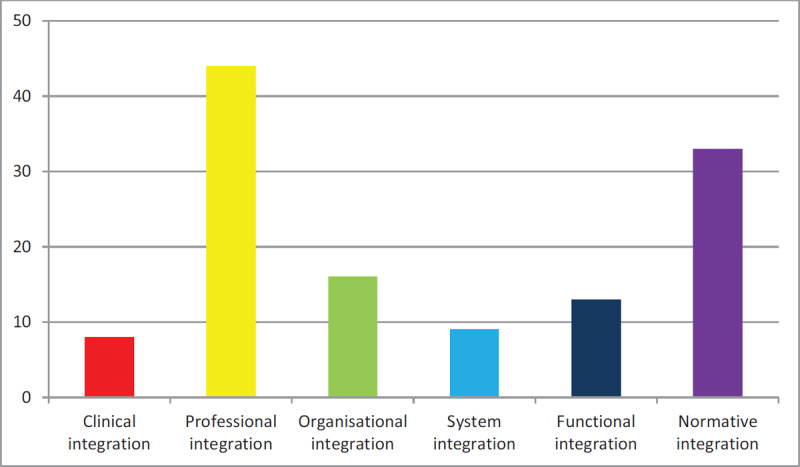
Match of Studies with Indicators from the Domains from the Rainbow Model (% of matching indicators).

**Rainbow model** [46,47]. The Rainbow model from Valentijn and colleagues was used to code the IPC characteristics from the empirical studies, including the items with final consensus from the international Delphi studies. We calculated a total score (% of all indicators) for each domain to determine the IPC focus of the studies (see Figure 2); inter-coder agreement was determined for the total scores of each main category with the intraclass correlation coefficients (ICC, two way mixed, absolute agreement). Agreement was adequate for the total scores for each domain: Clinical integration (ICC = .75); Professional integration (.73); Organisational integration (.78); System integration (.80); Functional integration (.76); and Normative integration (.81).

**Figure 3:**
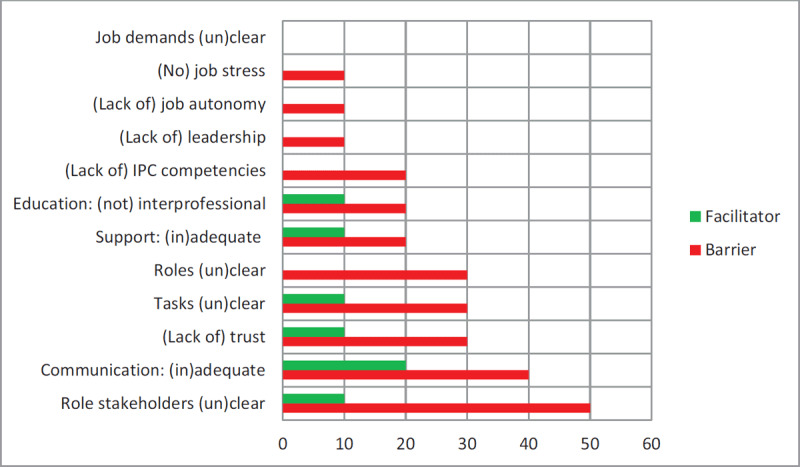
Frequency of IPC Themes for Included Studies as Barrier or Facilitator (% of studies).

**Context-Mechanism-Outcome (CMO) configurations** [28,31]. CMO configurations are a heuristic tool to explore mechanisms that are assumed to operate in IPC. They offer a contextualized understanding of causal mechanisms in an interprofessional team with contexts at societal, organisation, team or individual level. Identifying mechanisms may present a challenge in realist reviews [28], also because the original studies may not be designed as a realist evaluation with an explicit CMO framework. Full CMO configurations were sometimes formulated in one sentence or paragraph. However, it was also possible that different parts of a configuration could be identified in adjacent, but different parts of the report and were then combined in a full CMO sequence. This procedure was only used when the C, M and O parts were repeatedly mentioned at different places from the report, for example in the research questions from the introduction, the results or the conclusion section. Multiple contexts or multiple outcomes were possible for a specific mechanism if the authors explicitly linked multiple contexts and/or outcomes to a specific mechanism.

Initially, we distinguished between a micro, meso and macro level when we coded C, M and O. However, this categorization proved rather crude, also because the macro level appeared unimportant in our sample. We subsequently coded the level of C, M and O using the Gears model of Mulvale, Embrett, and Razabvi [48]. This model, which allowed a more differentiated coding of the configurations, distinguishes between six levels: the level of macro policy (indicated with 1), meso policy (2), micro team structure (3), micro team processes (both social and formal); 4), micro team attitudes (5) and, finally, the level of an individual professional (6). The model assumes that processes may occur either in a top-down fashion (i.e., from macro level to lower levels) or in a bottom-up fashion (i.e., from the individual level to macro). The Gear model takes into account cross-level interactions and a combination of different levels in a single CMO sequence is thus possible.

We complemented our coding by adding for each component whether C, M and O were framed in the report as intended-positive (‘+’) or unintended-negative (‘–’). A context may thus promote or hinder IPC; for example, role confusion was described as a factor that complicates effective IPC and was coded with ‘–’. Similarly, a mechanism in a study may foster or hinder IPC; for example, the resolution of a conflict may result into a better understanding of professional roles, and was therefore coded as a positive mechanism.

In sum, each identified sequence was coded with the general C_1-6,+/–_M_1-6,+/–_-O_1-6,+/–_ scheme (see Table 1). Both coders independently identified CMO configurations for each study and discussed their work extensively in multiple sessions. CMO configurations were cross-examined against the Gears framework to analyze whether the studies reported mechanisms with identical or adjacent levels. We also checked the consistency of configurations by focusing on the congruency between positive/negative elements; for example, a positive mechanism should theoretically be coupled with a positive outcome.

**Table 1:** IPC related CMO Sequences from the Included Studies.

Study (only 1^st^ author)	Setting	Context(s)	Mechanism	Outcome(s)

			**Mechanism related to macro policy [1]**	

–	–	–	NA	–
			**Mechanism related to meso policy [2]**	

Visscher (2017)	Primary and secondary preventive child healthcare	Variation within & between organisation policies (2, –) Different talents/training (3, –)	Implementing policy, funding, and training for interprofessional communication (2, +)	IPC (2, +) Medical expertise (6, +)
			**Mechanism related to team structure [3]**	

He (2017)	Child welfare system	Neglect of substance use disorder (2, –)	Co-location of multi-disciplinary staff (3, +)	Resources increased at organisational level (2, +)
O’Reilly (2011)	Child protection work	Effective inter-organisational communication (4-social, +) Role complexity (5, –) Workload documentation requirements (4-formal, –)	Lack of leadership (3, –)	Non-functional interprofessional team (4-social&formal, –)
Weglarz-Ward (2016)	Childcare (for children with disabilities)	Different levels of role uncertainty and confusion within and between professional groups (5, –) Childcare providers consistently ranked their benefits as lower than early intervention providers (6, –)	Planning interventions without childcare providers being formally included (3, –)	Unsuccessful professional collaboration and lack of inclusion (2, –)
			**Mechanism related to social team processes [4]**	

Simpson (2017)	Children integrated services	Limited time and resources (2, –) Policy reform, budget cuts (1, –)	Conflict resolutions (4, +)	Ability to employ creative solutions (4-formal, +)
Timonen-Kallio (2017)	Residential childcare	Fragmented context services (1, –) Unrealistic expectations and perceptions to support children (5, –)	Delivering expert knowledge rather than sharing responsibility (4, –)	Uncertainty about competences, responsibilities and authority (6, –)
			Taking ownership of the care plan, thereby excluding others (4, –)	Rely more on others’ expertise than on their own expertise (6, –)
			Social workers acting as mediator (4, +)	Less uncertainty & rely on one’s own expertise (6, +)
O’Reilly (2011)	Child protection work	Different preferred modes of communication (4-social, –)	Meeting the team face-to-face instead of using technologically driven modes of communication (4, +)	Less workplace stress (5, +) More job satisfaction (6, +) Recognition of one’s abilities (5, +)
			**Mechanism related to formal team processes [4]**	

Hood (2017)	Child safeguarding	Lack of mutual understanding: Holistic approach versus only one area (4-formal, –) Flexible versus detailed procedures (4-formal, –) Sound knowledge internal safeguarding versus external healthcare settings (4-formal, –) Pre versus post qualifying practice (3, –)	Conceptualizing practice (i.e., the requisite knowledge and awareness to do safeguarding work, joint assessments, and managing risk) (4, +)	Mutual understanding: between teacher and social worker about the role and methods of social worker (4-social, +)
		Teacher has long term relationships with parents vs. nurses and social worker have short term relationships (4-social, +) Social worker has authority role for parents (teacher not) (4-social, +) Conflict between teacher and social worker because parent plays them off against each other (4-social, –)	Managing relationships between parents and professionals (4, +)	Effective three-way relationship between social worker, teacher and parent (4-social, +)
Bolin (2017)	Special education (social workers, sex education for children with disabilities)	Role ambiguity in schools (4-social, –) Different value systems (5, –) Discomfort with topic (6, –)	Acknowledging and reducing role ambiguity in schools (4, +)	Feeling safe and comfortable (6, +)
Agresta (2018)	Social work in elementary, middle and high school	Role discrepancy (5, –)	Participating in informal and formal meetings (4, +)	Reduction of professional role discrepancy (5, +) Higher job satisfaction (6, +) Retaining staff (2, +)
			**Mechanism related to team attitudes [5]**	

Agresta (2018)	Social work in elementary, middle and high school	School social workers allocating professional time (4-formal, +)	Offering support and trust (5, +)	Higher level of job satisfaction (6, +) Retaining staff (2, +)
			**Mechanism related to individual staff [6]**	

Rose (2011)	Child and adolescent mental health services (special education needs)	Negotiation in multi-agency collaboration (4-formal, +)	‘Some kind of professional self-sacrifice’, related to professional identity, expertise, power and/or territory (6, +)	Individual staff enacts collective preferences (6, +)
Agresta (2018)	Social work in elementary, middle and high school	School social workers allocating professional time (4-formal, +)	Stimulating self-perceived autonomy of professionals (6, +)	Reduction of professional role discrepancy (5, +)

### Peer Review

The results were presented and discussed with a group of external stakeholders at a meeting from the national PACT expertise group (December 9, 2019). Chaired by the Dutch Kinderopvangfonds as an external funding body, this multi-disciplinary group with stakeholders from universities (University of Amsterdam, Utrecht and Nijmegen), universities of applied sciences (Amsterdam University of Applied Sciences, Hanze Hogeschool), expertise centers (Dutch Youth Institute, Kohnstamm Institute, Praktikon) and managers and other staff, shares an interest in interprofessional working and integrated child services and has attended meetings for four years. We handed out the figures and tables from the review to all participants and took minutes during the discussion to guide further reflection on our findings. At a second meeting at the University of Amsterdam (January 20, 2020), the results were presented and discussed with all members from the Preventive Youth Care master of the Research Institute of Child Development and Education.

## Results

All studies reported a descriptive study using a quantitative (60%) and/or qualitative method (80%); mixed-methods designs (30%) were also included. All studies had a single wave of data collection, including interviews (60%), surveys (40%) and/or focus groups (40%). Professionals were the informants in each study; no children or parents who ‘voiced’ their experiences were included. In total, the studies included 2,572 participants with significant variation between studies (*SD* = 423.7, *min-max*: 8–1105). Their work experience ranged from 5 years to 23 years. Women predominated in the study samples; not all studies reported demographic information, however.

The study participants worked in childcare (1 study), primary school (5x), social work (6x), or youth care (6x). The professional context was a universal, primary care setting for children (60%) and/or a special care setting for a targeted population (70%); the percentages do not add up to 100% because in some studies professionals from both settings participated. The professional roles of the staff [10] were mostly supportive and nurturing (50%), followed by assessment and monitoring (30%), health education and promotion (20%) and resources and referral (10%).

As Figure 2 shows, professional integration (PI) from the Rainbow model was the most frequent focus of the studies, followed by normative integration. Often recurring indicators of the professional integration dimension were ‘agreements on interdisciplinary collaboration’ and ‘interprofessional governance’. The indicator ‘linking cultures’ from the normative integration dimension was included in nearly all studies. The other dimensions of IPC from the Rainbow model were less common.

### IPC Barriers and Facilitators

Figure 3 indicates how many studies addressed the selected IPC themes, distinguishing between facilitators and barriers. Discussion of barriers in the studies predominated discussion of facilitators, reflecting a critical perspective on current IPC; the distribution of barriers/facilitators was 3.86 to 1, respectively. Specifically, lack of communication and distrust between the professionals from different disciplines was an important theme. Conversely, other studies emphasized the positive role of communication between various professionals. Many studies discussed unclear roles and tasks in the interprofessional teams; this is in agreement with the findings from the Rainbow model, where role ambiguity was a frequent indicator.

### CMO Configurations

The CMO configurations are listed in Table 1, ranked from the highest macro level to the lowest individual level. No mechanisms at macro level were identified in the included studies, but all other levels were present. Seen from the perspective of the Gears framework, the context, mechanism, and outcomes were often at identical or adjacent levels (i.e., C_X_M_C±1_,O_M±1_), although there were also configurations at divergent levels (i.e., C_X_M_C±2_,O_M±2_). In some cases, the difference was larger than two levels. The identified CMO configurations were internally consistent, as expected, looking at the sign of the mechanism and outcomes: positive mechanisms were consistently linked with positive outcomes, whereas negative mechanisms were linked with negative outcomes.

The analysis of the sign of the CMO configurations (‘+’ or ‘–’) revealed three patterns. Firstly, contexts were more often ‘negative’ than ‘positive’ (19x, 76% vs. 6x, 24%, respectively), which reflects that often unfavorable or challenging working conditions for IPC were emphasized in the study reports. Secondly, positive mechanisms outnumber negative mechanisms (12x, 75% vs. 4x, 25%) and, relatedly, outcomes were more often positive than negative (18x, 82% vs. 4x, 18%). Two configurations with a focus on IPC related difficulties consisted only of negative elements, whereas two configurations with a positive perspective included only positive elements. Most configurations (12, 75%) were mixed, however, and included both positive and negative C, M and O elements. A number of studies, for example, addressed a currently challenging context for IPC with barriers at one or more levels, and linked this with a positive mechanism to arrive at more favorable IPC outcomes. Finally, Table 1 shows a mixed pattern of both positive and negative findings at the level of team structure and team processes (both social and formal), whereas contexts, mechanisms and outcomes are uniformly positive for team attitude and individual staff. Hence, positive contexts, mechanisms and outcomes were more frequent in the reviewed studies, moving from the highest level to the lowest level of individual staff.

The studies addressed various concrete contexts, mechanisms and outcomes related to IPC. Important and often challenging contexts for IPC are fragmented youth services, different policies of the organisations ‘around the child’, together with limited resources and policy reforms. In addition, individual staff may face high workloads or unrealistic expectations. At team level, professionals may experience uncertainty about their roles due to different working procedures from staff with different disciplinary backgrounds. An important context is that regular staff like caregivers and teachers have daily, long-term relationships with children and parents, whereas specialized staff have short-term relationships with the families [38]. Both professionals have thus fundamentally different relationships with the child and/or the parents. A further difference is that professional caregivers and teachers generally focus on the broad development of the child and are responsible for all children in a group setting, whereas specialized staff focus more on a specific part of the development of children (e.g., sexual development), often for a selection of the children in a regular setting. Finally, regular staff may perceive their professional role as less important than the role of specialized staff.

The identified mechanisms involved both barriers and facilitators of IPC. Barriers that frustrated collaboration in IPC practice at the level of team structure are a lack of leadership with regard to the collaboration of professionals with various backgrounds and excluding others in the planning of interventions [see also 22]. Conversely, designing a policy for interprofessional collaboration and providing a training for all team members were assumed to contribute to IPC. These mechanisms point at the importance of linking an IPC vision at organisation level with staff training for the team. The organisation management should also formally designate professionals to make clear who is involved in working with a child and the parents. At team level, the social process of collaboration may be impeded by colleagues who take ownership of plans (vs. sharing) or deliver knowledge but do not take responsibility during interprofessional meetings. Also, different opinions about the modes of communication (e.g., face-to-face, phone, email) may hinder collaboration at team level. Conversely, co-location of staff and organising formal and informal face-to-face meetings were identified as positive mechanisms. These identified mechanisms strongly suggest that professionals prefer a non-hierarchical way of collaboration with equal responsibilities and shared modes of communication in the planning and execution of their work. Conceptualizing practice, conflict resolutions and reducing role ambiguity, possibly facilitated by one of the team members in a mediating role, was also considered to facilitate IPC at team level.

At individual level, some professionals experienced a difference in power and professional status. Specifically, teachers from regular childcare and school felt that their role was considered to be less important than specialized staff, like social workers or early intervention specialists. Further, IPC may require from staff at individual level ‘some kind of professional self-sacrifice’ [19], which results in enacting a collective preference, according to Rose [38], whereas Agresta [33] asserts that stimulating the self-perceived autonomy of professionals results in a reduction of professional role discrepancy. This suggests that professionals should strike a balance between self-sacrifice and self-perceived autonomy in interdisciplinary collaboration.

At the first peer review session, the participants generally expressed recognition of the findings from the reviewed literature. They indicated that professional integration and normative integration are often predominant in the very first stage of interprofessional collaboration. New relationships are built in this stage and the focus of individual staff may be on professional integration and the importance of shared professional values as part of normative integration. The researchers at the session also recognized the focus on interprofessional collaboration in current research. In fact, research (also in the Netherlands) has focused more on professional integration than on clinical integration. The focus on challenging contexts for IPC, as indicated by our review, was also recognized by the researchers. Based on their experience, the practitioners emphasized however that the stage of IPC influences professionals’ experiences. In the beginning, staff is often exploring own and others’ professional roles in a new group dynamic with possible tensions between team members. In addition, IPC may not be fully effective yet at this early formation stage, which means that professionals invest in interdisciplinary collaboration without seeing concrete results in their daily practice. These experiences during the early stages were certainly not typical for IPC teams from a long-term perspective, however. The practitioners highlighted that IPC had resulted for them into a more flexible team where individual staff with complementary competencies share the responsibility for children’s broad development. In short, the researchers emphasized an ongoing shift from professional and normative integration to client integration (from ‘moving from yellow to red’, as one of the participants said, referring to Figure 2), whereas the practitioners underlined the importance of distinguishing stages in team development (‘moving from red to green’ in Figure 3). At the second meeting at the University of Amsterdam (January 2020), participants suggested, from an academic perspective, that an elaborated framework may be beneficial in categorizing contexts, mechanisms and outcomes. We further discussed whether realist synthesis is primarily aimed at generating, refining or testing hypotheses. Our synthesis seems mostly focused on generating and refining hypotheses.

## Discussion

Our review gives insight into the experiences and perceived barriers and facilitators of professionals with special expertise collaborating with staff from regular settings in community care. The reviewed studies, which foregrounded the experiences and perceptions of primary staff, underline that professional integration is often complicated for both specialized staff and staff who collaborate in regular community-based settings. Our review revealed a significant imbalance in the barriers and facilitators of IPC (see Table 1 and Figure 3). Specifically, the professionals involved in IPC experienced unclear professional roles in IPC context. Excluding others in the planning of interventions, taking ownership of plans, different modes of communication, and feelings of distrust were identified as barriers for effective IPC. Important facilitators were interprofessional training, co-location of staff, organising formal and informal meetings at organisation level [49]. At team and individual level, conceptualizing practice, self-sacrifice, conflict resolutions, and acting as a mediator in the team were considered important facilitators of IPC (see Table 1). A general finding from our review is that contexts are challenging, particularly at the higher levels from the Gears framework. A challenging context at team level is that different professionals have fundamentally different relationships with children and their parents: regular staff have long-term relationships with families and often meet on a daily basis, whereas specialized staff have less contact with children and parents; see also [50] for a related pattern in health care with nurses vs. specialized staff.

The reviewed studies share a focus on professional and normative integration. Clinical integration and integration at organisational, system or functional level proved minor themes (see Figure 2). Our findings suggest that many professionals in the included studies were finding their way with collaborating with other professions. They were becoming familiar with each other, and, relatedly, the levels of IPC seemed at an intermediate, but certainly not high level. The fact is that close cooperation between early childhood education and care and school with other sectors like youth care and social work has existed at different places but is not commonplace in various countries and may even be a relatively new phenomenon for many practitioners [5]. This relatively early stage of IPC may also partially explain why attention to team building [51] was a dominant theme in the literature, coupled with crossing disciplinary boundaries by team members, role release, and conflict management skills. The findings from our review suggest that communication, trust and strong interpersonal relationships need to develop before professionals start building consensus, making team decisions and sharing leadership. This finding fits in with the public health literature, which also stresses the vital importance of interdependent collaboration, open communication and shared decision-making at team level [52].

## Strengths and Limitations

Our review has revealed a number of barriers, facilitators and mechanisms within the domain of professional and normative integration for IPC for regular and specialized staff in children’s services. Our coding of the studies showed convergence, which supports the validity of our findings.

Our extended coding system for the CMO configurations proved valuable in both identifying and interpreting the mechanisms from the studies in our review. Although identifying CMO mechanisms remains a challenge for reviewers [28], customizing the configurations with the Gears framework and indicating the sign of each context, mechanism and outcome proved feasible in our review. Possibly, our coding scheme may also be helpful in other realist syntheses. For some configurations, however, there still seems to be a conceptual distance between context, mechanism and outcome, also because some outcomes were formulated in rather general terms (i.e., ‘job satisfaction’, ‘retaining staff’ or ‘effective IPC’). The Gears metaphor from Mulvale and colleagues [48] suggests that context, mechanism and outcomes are at identical or adjacent levels with each other, and future studies may explore whether CMO configurations with closer distances between context, mechanism and outcomes are theoretically more coherent and receive more empirical support.

From a methodological perspective, it should be noted that the reviewed studies relied heavily on self-reports. This line of research has resulted into a vivid and often detailed picture of individual professionals’ experiences and their interdisciplinary practice. However, the lack of triangulation of methods and informants limits the validity of the included primary studies, and, relatedly, we could not ascertain in our review whether there is a possible bias due to self-reports. The reliance on self-report measures may also have influenced our CMO analysis. Moving from the macro level to the team level and, finally, the individual practitioner level from the Gears framework, the included studies reported increasingly more positive mechanisms and outcomes. This finding may reflect the existence of a relatively large number of barriers at meso and macro levels, compared to individual and team level. However, it is also possible that participants are more likely to identify facilitators when they reflect on individual competencies and team members from their immediate working environment (i.e., resulting in relatively positive perceptions at lower levels from the framework), while they tend to focus more on barriers at meso and macro levels because they are beyond their purview (i.e., resulting in less positive perceptions at these higher levels).

The included studies from this review mainly focused on a variety of professional caregivers in (mostly) universal child services. The integration across universal child services like early childhood education and care and primary schools, community-based health care and specialized health care may foster positive outcomes for families and may also promote job satisfaction of professionals. This important topic deserves more attention and future research may bridge the gap between IPC in regular, community-based settings and IPC in specialized healthcare systems.

## Conclusion

Does IPC “work” in the context of regular children’s services, and for whom and under what circumstances? Our review suggest that IPC may work for generalist and specialized staff who aim to offer integrated services for families with young children, acknowledging that moving together towards a new system is fraught with challenges for staff. At this stage in both practice and research, we know yet little of what IPC may offer to children. Future studies should therefore include client-reported measures for youth and their families to investigate their experiences with IPC. Facilitators for IPC in this specific context are crossing disciplinary boundaries, shared decision making and interprofessional training. Strengthening teams and a focus on interpersonal relationships and building trust also supports IPC. The identified barriers and facilitators from this review are all related to a defining feature of IPC in community-based children’s services: generalists who interact with children on a daily basis to stimulate their holistic development in a group context meet in their new collaboration specialists who often focus on specific parts of children’s development, work outside the regular classroom, and have different working relationships with children and families. The implementation of IPC should not aim to unify these professionals with diverse backgrounds, but should provide staff efficient ways to offer personalized support for young children in their early years.

Research into IPC shows a dominant focus on staff with various disciplinary backgrounds and their professional and normative integration in community settings. Obviously, the study of the complex variable IPC in its own right is valuable for research and practice. However, clinical integration seems currently a largely unexplored territory and we know relatively little about the relationship between IPC and children’s development and parents’ experiences [53]. Future research should bring together staff from community services, professionals with special and complementary expertise, and children and their families. Finally, a shift from descriptive studies to longitudinal or experimental studies would provide additional evidence for the hypothesized causal mechanisms that foster effective IPC.

## Appendix A

Key Words from Literature Search.

**Table:**

Interprofessional collaboration domain	Professionalism domain	Practitioners domain	Goal domain

collaboration/ OR cooperation/ OR (alliance* OR coalition* OR collaborat* OR cooperat* OR co-operat* OR intraorgani*ational OR intraprofessional OR interdiscipl* OR interorgani*ational OR interprofessional* OR multidiscipl* OR partnership* OR transdiscipl*).ti,ab,id.	professional development/ OR professional competence/ OR professionalism/ OR (expertise OR skill acquisition* OR more proficient OR professionali*ation OR professional development).ti,ab,id.	child care workers/ OR social workers/ OR (practitioner* OR worker* OR caseworker* OR professionals OR teacher* OR ((child care OR youth OR youth care OR youthcare OR child welfare OR social OR frontline) ADJ3 (coach OR coaches OR professional OR team* OR staff OR personnel))).ti,ab,id.	((infan* OR baby* OR babies OR toddler* OR preschool* OR child* OR kid OR kids OR prepubescen* OR prepuberty* OR teen* OR young* OR youth* OR girl* OR boy*) AND (safeguard* OR protect* OR child care OR child welfare OR infant welfare OR social casework* OR social case work* OR social work* OR social services OR youthcare OR youth care OR youth work*)).ti,ab,id.

*Note*: ti = title; ab = abstract; id = identifier. ADJ3 = adjacent within 3 words.
